# Chemical and Nutritional Characterization of Sourdoughs Made with Sprouted and Unsprouted Whole-Wheat Flour and Their Effects on the Technological Quality of Bread †

**DOI:** 10.3390/foods14162805

**Published:** 2025-08-13

**Authors:** José Luis Navarro, María Soledad López, Emiliano Salvucci, Alberto Edel León, María Eugenia Steffolani

**Affiliations:** 1 Instituto de Ciencia y Tecnología de los Alimentos Córdoba (ICyTAC), CONICET—Universidad Nacional de Córdoba (UNC), Córdoba CP 5000, Argentina; josenavarro@agro.unc.edu.ar (J.L.N.); mslopez@agro.unc.edu.ar (M.S.L.); esalvucci@agro.unc.edu.ar (E.S.); eusteffolani@agro.unc.edu.ar (M.E.S.); 2 Facultad de Ciencias Agropecuarias, Universidad Nacional de Córdoba (UNC), Córdoba CP 5000, Argentina

**Keywords:** sprouted whole-wheat flour, sourdough, bioactive compounds, whole-wheat bread, technological quality, volatile organic compounds

## Abstract

There is a growing interest within the food industry sector in applying natural and sustainable methods to improve functional, nutritional, and technological properties of foods. In this regard, sprouting and spontaneous sourdough (SD) fermentation, are emerging as promising technological solutions in the bakery industry. The aim of this work was to evaluate the effect of using unsprouted whole-wheat flour (USWF) and sprouted whole-wheat flours (SWFs), obtained under controlled conditions (20 and 25 °C for 24 h), on the chemical and nutritional properties of spontaneous SD and their impact on whole-wheat bread technological quality. SDs were prepared with a dough yield of 200, incubated at 30 °C for 24 h, and refreshed daily for 7 days. In general, an increase in both yeast and lactic acid bacteria counts was observed in all SD samples. All SDs showed reduced α-amylase activity and enhanced contents of free amino acids groups, water-extractable arabinoxylans, total phenolics, and antioxidant capacity, along with lower phytic acid content. Substituting 20% of USWF with SD improved bread volume and crumb softness. Notably, breads made with sourdough prepared from 20% sprouted whole-wheat flour (SWF25) promoted the formation of volatile compounds associated with pleasant aromas, which may increase consumer acceptability. Promising nutritional and sensory advantages could result from combining fermentation and sprouting.

## 1. Introduction

In recent years, consumer awareness of health and nutrition has grown significantly, driving demand for natural and functional ingredients. Consequently, there is growing attention to sustainable and practical processing methods to enhance the nutritional and functional properties of whole grains while minimizing their technological limitations in baked goods. In this context, traditional and well-established practices such as controlled sprouting and sourdough fermentation are gaining renewed attention as innovative approaches in the baking industry.

Sprouting is a biotechnological process that initiates the transformation of seeds into seedlings, leading to physiological and biochemical changes. These include the activation of endogenous hydrolytic enzymes, which enhance the nutritional quality and digestibility of wheat proteins and starch [1]. The use of sprouted whole-wheat flour as a nutritious and techno-functional ingredient has been well documented [2,3,4]. A previous study by Navarro et al. [2] proved that sprouting activates the grain’s biosynthetic potential, increasing the content of reducing sugars, free amino acids, soluble fibers, and phenolic compounds through enzymatic hydrolysis.

Sourdough fermentation, one of the oldest biotechnological processes, also affects several quality attributes of baked goods through the metabolic activity of autochthonous lactic acid bacteria (LAB) and yeasts [5]. Traditionally used as a leavening agent, sourdough is now valued for reducing additive use in breadmaking [6] and for its technological and nutritional benefits, such as improved rheology [7,8]. These effects are linked to microbial metabolite production, antinutrient degradation, enhanced protein digestibility, and lower glycemic index [9]. Furthermore, sourdough generates more volatile compounds than yeast-only fermentation, improving sensory quality [10,11].

The fermentation capacity of sourdough depends not only on microbiota composition and fermentation conditions but also on the type of flour used [12]. Notably, wheat enzymes supply crucial substrates for microbial metabolism. These endogenous enzymes influence LAB activity, particularly in carbohydrate, protein, phenolic, and lipid metabolism [6]. Using sprouted whole-wheat flours as fermentation substrates may impact both the composition of the sourdough microbial community and the resulting metabolic activity. While sprouted grains and sourdough fermentation have been individually characterized for their nutritional, functional, and sensory benefits, the combined effect of using sprouted whole-wheat flour in spontaneous sourdough systems remains underexplored. The enzymatic products released during controlled sprouting, such as reducing sugars and free amino acids, could influence the development of sourdough microbiota. This may alter LAB-to-yeast ratios, shift the balance between homo- and heterofermentative LAB species, and reshape microbial metabolic interactions. These changes could ultimately affect the chemical composition of sourdough and the technological quality of the final baked product, including specific bread volume, crust and crumb color, and texture profile [13].

The aim of this study was to investigate the effects of traditional Type I sourdough fermentation using unsprouted and sprouted whole-wheat flours obtained under controlled sprouting conditions. Using a back-slopping protocol and a dough yield of 200, we evaluated changes in the chemical and nutritional composition of the flours and sourdoughs, as well as the impact of a 20% substitution of whole-wheat flour with sourdough on the technological quality parameters of bread and its volatile organic compound profiles.

## 2. Materials and Methods

### 2.1. Materials

We used the commercial wheat variety Klein Valor (*Triticum aestivum* L.), provided by INTA’s Estación Experimental Agropecuaria Marcos Juárez (Córdoba, Argentina), and grains from the same lot sprouted in the laboratory under controlled conditions. Baker’s dry yeast (Saf-Instant, Lesaffre, Toluca, México) was obtained from the local market. All chemicals and solvents were analytical grades (Cicarelli, San Lorenzo, Santa Fe, Argentina). Whole-wheat flour consisted of moisture (%) 10.46 ± 0.09, ash (%, db) 1.59 ± 0.03, protein (%, db) 12.03 ± 0.03, lipid (%, db) 2.22 ± 0.05, and starch (%, db) 67.2 ± 0.4. Each determination was assessed according to AACC approved methods 44–15.02, 08–01.01, 46–12.01, 30–26.01, and 76-13.01, respectively [14].

### 2.2. Methods

#### 2.2.1. Sprouting Conditions

The wheat sprouting conditions were selected based on preliminary tests [2]. Briefly, wheat grains were steeped in drinking tap water at 18 °C for 24 h (1:2 water ratio). After draining, the grains were placed in trays and incubated in a germination chamber (Servicios Mecatrónicos, Córdoba, Argentina) at 20 and 25 °C with 95% relative humidity in the dark. Samples were collected after 24 h for analysis. The degree of sprouting (DoS) of 100 grains was visually assessed based on coleoptile and radicle length (0–7 degrees of sprouting) following Krapf et al. [15]. The sprouted grains were then dried in an air-circulating oven at 50 °C for 20 h to limit water availability for enzymatic activity. Finally, the sprouted grains, including vegetative parts, were milled using a cyclonic mill (Cyclotec™ 1093; Foss, Hillerød, Denmark) with a 1 mm mesh sieve to obtain sprouted whole-wheat flours (SWF20 and SWF25), corresponding to flours from grains germinated at 20 °C and 25 °C, respectively. Unsprouted whole-wheat flour (USWF), processed under the same milling conditions was used as a control.

#### 2.2.2. Sourdough Preparation

The fermentation process was conducted as a traditional Type I sourdough in duplicate under sterile conditions by mixing USWF or SWFs with water in a 1:1 ratio to produce a dough yield (DY) of 200 (dough weight/flour weight × 100). The flour–water mixtures were incubated at 30 °C through propagation by back-slopping 20% of the previously fermented dough with a new mixture of flour and water until a stable microbiota was established (7 days). SD samples were collected at 0 h (t0), corresponding to the dough prior to fermentation, and subsequently after 7th refreshment (t7). Finally, part of the ripe sourdoughs was used in the bread-making process, while the remaining amount was frozen at −40 °C for subsequent freeze-drying and chemical analysis.

#### 2.2.3. pH and Total Titratable Acidity and Microbial Count

The pH of SD samples was determined with a pH meter for semisolid compounds TESTO 205 (Testo Lenzkirch, Germany). The acidification capacity was expressed as ΔpH, calculated as the difference between initial and final pH values [16]. Total titratable acidity (TTA) was determined following Lancetti et al. [7]. For microbiological analysis, 10 g of SD samples was homogenized in 90 mL of sterile 0.9% (*w/v*) NaCl solution and subjected to serial decimal dilutions (10^−1^ to 10^−6^). Lactic acid bacteria (LAB) were quantified on De Man, Rogosa, and Sharpe (MRS) agar (Oxoid, Basingstoke, Hampshire, UK) supplemented with cycloheximide (0.1 g/L) under anaerobic conditions at 37 °C for 48 h. Yeasts were assessed on using Yeast Glucose Chloramphenicol Agar (YGC) (Sigma-Merck, Darmstadt, Germany), with plates incubated aerobically at 25 °C for 48 h. All the analyses were carried out in triplicate.

#### 2.2.4. Chemical Analysis of Flour and Freeze-Dried Sourdoughs

##### α-Amylase Activity

The α-amylase activity was measured using the Amylazyme method (Megazyme, Bray, Ireland). Samples (0.2 g) were incubated at 60 °C in sodium maleate buffer (pH 6.0) with CaCl_2_, then reacted with an Amylazyme tablet. The reaction was stopped using Tris solution (pH 9.0), and the mixture was filtered. The absorbance was measured at 590 nm with UV-Vis spectrophotometer (model V-730, Jasco Co., Tokyo, Japan). Activity was calculated from the standard curve supplied with the Amylazyme kit (Megazyme) and expressed in α-amylase units (AU) per gram (db). Determinations were performed in triplicate.

##### Reducing Sugar Content

The reducing sugar content was determined by the 3,5-dinitrosalicylic acid (DNS) method described by Bustos et al. [17]. Absorbance was measured at 540 nm with a UV-Vis spectrophotometer (model V-730, Jasco Co., Tokyo, Japan). Glucose was used for calibration, and the results were expressed as g of glucose per 100 g of sample (db). Determinations were performed in triplicate.

##### Free Amino Acid Content

The number of peptide bonds cleaved by endoprotease activity was indirectly assessed based on the amount of free amino groups using the o-phthalaldehyde (OPA) method, as described by Perri et al. [13]. Absorbance was measured at 340 nm with a UV-Vis spectrophotometer (model V-730, Jasco Co., Tokyo, Japan). The quantification of the results was carried out from a calibration curve using serine as a standard. The results were expressed as μmol serine per mg protein (db). Determinations were performed in triplicate.

##### Water-Extractable Arabinoxylans Content

The content of water-extractable arabinoxylans was determined using the Orcinol-HCl method described by Lancetti et al. [7] at 670 nm with UV-Vis spectrophotometer (model V-730, Jasco Co., Tokyo, Japan). The results were expressed as g WEAX/100 g flour (db). Determinations were performed in triplicate.

##### Total Polyphenol Content and Antioxidant Capacity

Polyphenols were extracted as described by Bustos et al. [17], by mixing 500 mg of sample with 5 mL of an acetone–water (70:30) solution containing 0.1% HCl for 6 h in the dark. Total polyphenol content of the extracts was determined using the Folin–Ciocalteu method adapted by Bustos et al. [17]. Gallic acid was used to prepare the standard curve, and the results were expressed as mg of gallic acid equivalents per 100 g of sample (db). Antioxidant capacity was evaluated through reducing activity using the ferric reducing antioxidant power (FRAP) assay, and through free radical scavenging activity using the ABTS˙^+^ assay, according to Podio et al. [18]. Absorbance was measured at 593 and 734 nm, respectively, with a UV-Vis spectrophotometer (model V-730, Jasco Co., Tokyo, Japan). The quantification of the results in both methods was performed using a calibration curve, using Trolox as standard. The results were expressed as µmol of Trolox equivalents per 100 g (db). Determinations were performed in triplicate.

##### Phytic Acid Content

The phosphorus content of phytic acid was determined following the Haug and Lantzsch photometric method modified by Raboy et al. [19]. Absorbance was measured at 519 nm with a UV-Vis spectrophotometer (model V-730, Jasco Co., Tokyo, Japan). Dodecasodium phytate was used for calibration, and the results were expressed as mg of available phosphorus phytate per g of sample (db). Determinations were performed in triplicate.

#### 2.2.5. Breadmaking Procedure

Breadmaking tests were performed according to Steffolani et al. [20]. The control bread (CB) was prepared using 400 g of USWF. To make sourdough-substituted breads, 20% of the USWF was substituted with ripe SD (USWF) or SD (SWF20 and 25). The substitution level of whole-wheat flour with sourdough used in the bread formulation were selected based on preliminary tests conducted by the same research group [21]. Additionally, dry yeast (12 g) and sodium chloride (8 g) were used for dough preparation. The amount of water added to the bread formulation was previously determined experimentally. The ingredients were mixed with a planetary mixer (Peabody SmartChef, Buenos Aires, Argentina). The dough was allowed to rest for 30 min at 35 °C and 96% relative moisture, then sheeted in a Mi-Pan vf roller (Mi-Pan, Córdoba, Argentina), divided into 3 pieces (200 g), and molded into loaf shapes (Braesi MB 350, Caxias do Sul, Brazil). The loaves underwent a second proofing under the same conditions until reaching their maximum volume increment (75 min). Finally, the loaves were baked at 215 °C for 15 min in a Beta 107 IPA oven (Pauna, Córdoba, Argentina). Breadmaking was performed in duplicate. The loaves were stored in sealed polyethylene bags in a temperature-controlled room (25 °C) for 72 h.

#### 2.2.6. Bread Technological Quality

Bread volume was determined 2 h after baking by rapeseed displacement. The specific bread volume (SBV) was determined after baking as the bread volume/mass ratio (mL/g) according to method 10-05.01 [14]. Bread crumb and crust chromatic characteristics were measured based on the CIELab system with a Minolta 508d colorimeter (CR-400, Konica Minolta, Tokyo, Japan), defining three axes: L* (brightness), a* (red–green), and b* (yellow–blue). A browning index (100 L*) was also calculated [22]. Additionally, two central slices (15 mm thickness) were selected from each bread for crumb moisture, water activity, and texture profile. The crumb water activity (a_w_) was measured at 25.0 °C using an Aqua-Lab Water Activity Meter (208 Series 3, Decagon Devices Inc., Pullman, WA, USA). The crumb texture profile analysis (TPA) was performed in triplicate using an Instron Texture Analyzer (Universal Testing Machine, Instron Model 3342, Canton, OH, USA) with a 25 mm diameter probe at a speed of 5 mm/s and 40% compression at different time intervals (2, 24, and 72 h). To determine the effect of storage time in polyethylene bags at room temperature, the firming rate was calculated as the slope of the line derived from the regression analysis of the three data points on a force-time plot [7].

#### 2.2.7. Determination of Volatile Organic Compounds in Breads

For the analysis of volatile organic compounds (VOCs) of bread crumb and crust, the method described by [23] was followed. CB, SB (USWF), and SB (SWF25) (24 h after baking) were ground, and 1.5 g were weighed in duplicate into 10 mL headspace vial. Solid-phase extraction (SPME) and gas chromatography (GC) were performed using a CP-3800 system (Varian, Palo Alto, Santa Clara, CA, USA) with a Saturn 2200 detector (Varian, Palo Alto, USA), automatic PAL injector (Varian, Palo Alto, USA) with an Agilent No. 122-7032 DB-Wax column, 30 m × 0.25 mm (Santa Clara, CA, USA). A divinylbenzene/carboxen/polydimethylsiloxane 50/30 mm fiber (Supelco, Sigma-Aldrich, Allentown, PA, USA) was used. The VOCs adsorbed onto the fiber were thermally desorbed for 10 min at 260 °C. Helium (1.7 mL/min) was used as the carrier gas, and the injector was in splitless mode for 10 min using a 0.75 mm ID inlet liner. The detector temperature was 200 °C and, for ionization, used an electronic energy of 70 eV. The column temperature was programmed from 40 °C to 220 °C, with a total cycle time of 35.5 min. Volatile compounds were identified by comparing their spectra and retention indices with those in the NIST05 library (NIST, Gaithersburg, MD, USA) [7]. The mass spectra were analyzed using the Varian MS Workstation software (Version 6.6). The contents of the volatile compounds were expressed as the relative peak area counts of the compounds identified (peak area of each compound/total area × 100). Determinations were performed in duplicate.

#### 2.2.8. Statistical Analysis

Results are expressed as the mean of at least two replicates. Statistical analysis of data was conducted using InfoStat software (InfoStat, Cordoba, Argentina, Version 2022). An analysis of variance (ANOVA) was performed at a significance level of 0.05, using the DGC multiple comparison test to assess the significant effects of sprouting and fermentation on the response variables.

## 3. Results and Discussion

### 3.1. Effect of Sprouting Conditions on the Degree of Sprouting

After 24 h, differences in the progress of sprouting at both temperatures were clearly visible. When sprouting at 20 °C, the average DoS was 2.14, with over 80% of the grains exhibiting a DoS of 2. The sprouting process was more pronounced at 25 °C, as this temperature favored an increased metabolic rate in the grains, leading to more homogeneous sprouting. Approximately 90% of the grains reached a DoS of 3, characterized by the presence of a well-developed embryo emerging from the seed coat. It is worth noting that grains, even at this sprouting stage, are still considered whole grains [24].

### 3.2. Chemical Composition of Flour and Sourdough

This study provided a comprehensive evidence that controlled sprouting (20 °C and 25 °C) significantly influenced both biochemical composition and techno functional behavior of the resulting sourdoughs. As expected, grain metabolic activity increased with sprouting temperature, with the most pronounced modifications occurring in SWF25. Although α-amylase activity in SWFs increased by 25- to 30-fold compared to USWF, the resulting increase in reducing sugar content was limited to up to 4-fold (Table 1), likely due to the localized distribution of these enzymes in specific grain tissues [25]. A 1.5- to 2-fold increase in the content of water-extractable arabinoxylans in SWFs was also observed (Table 1). This rise could be attributed to the activation of cell wall-degrading enzymes, such as endoxylanases, during sprouting, although to a lesser extent than α-amylases [26]. Similarly, protein hydrolysis in SWFs, indirectly assessed by the content of free amino groups, also increased to a comparable extent relative to USWF (Table 1).

Table 2 presents the pH, TTA, and microbiological counts of yeasts and LAB for the SD samples. As expected, after 7 days of fermentation with daily refreshments, a decrease in pH and an increase in TTA were observed (Table 3). Ripe sourdoughs prepared with SWFs exhibited slightly higher pH values than SD (USWF). However, no significant differences were observed in TTA values among the SD samples. The use of SWFs as a substrate led to sourdoughs with a lower acidification capacity (ΔpH), compared to those SD prepared with USWF. The lower ΔpH of SD (SWFs) may be attributed to the higher initial acidity of these flours at t0 (Table 2), likely resulting from lipolytic hydrolysis during sprouting [27]. Similar results were reported by Montemurro et al. [12], where a lower acidification capacity was observed when using sprouted wheat flour as a substrate in the preparation of sourdoughs, although inoculated LAB were used. Microbiological analysis of sourdoughs at t0 was consistent with the findings of Perri et al. [13]; SD (SWFs) exhibited higher yeast and LAB counts than SD (USWF) prior to fermentation, whereas no significant differences in cell counts were observed among the SWFs (Table 2). In ripe SD samples, an increase in both yeast and LAB cell densities was observed compared to t0. Among these, ripe SD (SWF20) exhibited significantly higher yeast counts than SD (USWF). By contrast, SD (SWF25) had the lowest yeast count which could be linked to the higher reducing sugar content found in this sourdough. Simonsón et al. [28] found that yeast growth in wheat sourdoughs increased with sucrose concentrations up to 3.5%, but higher levels slightly reduced growth, likely due to osmotic stress. Considering previous studies [2], in which sucrose was identified as the predominant sugar in SWFs, osmotic stress could potentially occur. The cell density of LAB was significantly higher in SD (SWFs). Several authors already observed higher LAB counts in sprouted grains than native grains [16], likely due to the increased availability of carbon and nitrogen sources, such as carbohydrates, polymers, amines, carboxylic acids, and amino acids, resulting from the sprouting process [13].

The assessment of α-amylase activity in SD samples made with SWFs as a substrate revealed significantly reduced amylolytic activity compared to USWF (Table 1). This decrease is consistent with literature reports indicating that α-amylase is inhibited at pH levels below 4.5, primarily due to the accumulation of organic acids, such as lactic and acetic acid, produced by LABs metabolism during sourdough fermentation [6]. Despite this inhibition, a 5- to 7-fold increase in reducing sugar content was observed in the SD samples compared to their respective flours used as fermentation substrates (Table 1). This suggests that enzymatic hydrolysis of starch still occurs, with α-amylase remaining active during the early stages of fermentation before each refreshment until the pH drops to inhibitory levels [7]. Furthermore, this increase in reducing sugars could also be attributed to the action of acid-tolerant enzymes such as glucoamylase [5]. However, once sourdough is incorporated into bread dough formulations, the pH increases due to dilution and buffering effects from other ingredients. Under these conditions, α-amylase may recover its activity and contribute to further starch hydrolysis during proofing and baking [6]. These results highlight the dynamic enzymatic behavior of sourdough fermentation and the potential for preserving key techno-functional properties in SWF-based sourdough systems.

Regarding arabinoxylans, the results obtained clearly illustrated that flour fermentation contributed to fiber solubilization, in accordance with data reported by Manini [29]. Overall, SD samples showed that content of water-extractable arabinoxylans was higher than those found in their respective flours prior to fermentation (Table 1). However, sourdough made with sprouted whole-wheat flours as a substrate exhibited a 40–50% higher content of WEAX compared to the SD (USWF) (Table 1). This increase may be attributed to the activity of endoxylanases present in whole-wheat flours, which, unlike α-amylases, exhibit optimal activity within a pH range of 3.5 to 5.5 [30]. Previous studies have also reported an increase in WEAX content as a result of endogenous endoxylanase activity in sprouted wheat grain [12]. The hydrolysis of insoluble arabinoxylans from bran tissue may improve dough rheological properties by increasing system viscosity. This, in turn, could enhance carbon dioxide gas retention during leavening and contribute to improved texture in the final baked product [31]. Additionally, due to their ability to increase viscosity, WEAX can reduce the rate of the digestion and absorption of carbohydrates, resulting in beneficial effects on postprandial glycemic and insulinemic responses [28,31].

Spontaneous fermentation led to a 2- to 4-fold increase in the content of free amino acid groups compared to the respective flours used as fermentation substrates (Table 1), likely due to acidification shifting the dough system’s pH toward the optimal range for various proteases. Montemurro et al. [12] reported a 10-fold increase in the content of free amino acid groups in inoculated sourdoughs prepared from wheat flour sprouted for 3 days at 16.5 °C, compared to non-fermented flour. The contents of free amino acid groups in SD (SWF20) and SD (SWF25) were 10% and 30% higher, respectively, than in SD (USWF) (Table 1). These results suggest a partial depolymerization of proteins, which may result from both primary proteolysis, driven by endogenous proteases active in sprouted whole-wheat flour, and secondary proteolysis, derived from intracellular peptidases produced by LABs during sourdough fermentation [6]. Structural changes in proteins could influence dough rheological properties and, consequently, the technological quality of the final baked product. Furthermore, such modifications may affect the formation of aroma precursors that contribute to the bread aroma profile after baking [32].

### 3.3. Selected Nutritional Characteristics of Flour and Sourdough

Sprouting led to a 1.5- to 1.7-fold increase in total polyphenolic content (TPC) of the SWF extracts compared to those from USWF (Table 3). These findings were further supported by the reducing activity and ABTS˙+ scavenging activity results (Table 3). As reported by other authors, the improvement in antioxidant capacity during sprouting could be attributed to the solubilization of bound and conjugated phenolic compounds [33,34]. This is a result of the activity of hydrolytic enzymes such as endoxylanases and cellulases, which degrade cell wall components. Phytic acid present in whole grains flours and baked products represents a significant anti-nutritional factor to consider. A 10% reduction in phytic acid (PA) content was observed in (Table 3), likely due to phytases activation that hydrolyze phytate into myo-inositol and inorganic phosphate derivatives, which suggests that the sprouting process may enhance mineral bioavailability [25]. Regarding bioactive compounds, sourdough extracts showed a 1.8- to 2.5-fold increase in TPC compared to the polyphenol extracts of their respective flours prior to fermentation, except for SD (SWF25) (Table 3). The SD (SWF20) and SD (SWF25) extracts exhibited a 15–25% higher total polyphenol content compared to the SD (USWF) extract. In general, the FRAP reducing activity of the polyphenol extracts was higher than their reducing activity (Table 3). FRAP values increased by 25–50% after sourdough fermentation, while ABTS˙+ values increased 1.7- to 2.7-fold. Notably, SD (SWF20) and SD (SWF25) demonstrated antioxidant capacities 15–25% higher than those of SD (USWF) extracts, as determined by both methods (Table 3). There is evidence that structural degradation of fiber components occurs during spontaneous sourdough fermentation, promoting the release and/or synthesis of different bioactive compounds [16]. Montemurro et al. [12] reported an increase in total polyphenol content and antioxidant capacity after fermenting sprouted wheat flour with lactic acid bacteria.

A reduction of 90–95% in phytic acid content was observed after fermentation, although no significant differences were found between SD samples prepared with different flour types (Table 3). Previous studies have shown that LAB fermentation can efficiently degrade phytate complexes through the activation of both endogenous and microbial phytases during sourdough fermentation, particularly when pH levels drop below 4.6 [35]. These findings suggest that the sourdough fermentation of whole-wheat flours, regardless of whether sprouting is applied, may enhance mineral bioavailability and improve protein digestibility.

### 3.4. Effect of Sourdough Fermentation on Leavening and Technological Quality of Breads

**Figure 1:**
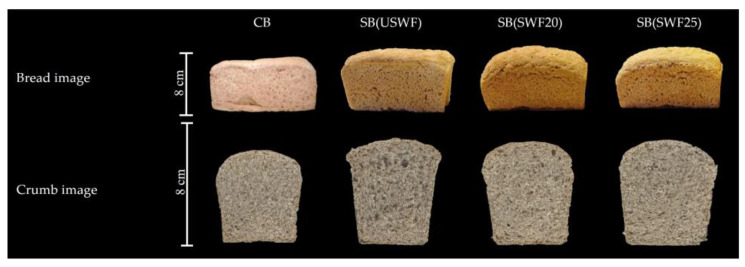
Representative images of whole-wheat breads. CB, control bread; SB (USWF), sourdough-substituted bread made with 20% sourdough from unsprouted whole-wheat flour; SB (SWF20 and SWF25), sourdough-substituted bread made with 20% sourdough from sprouted whole-wheat flours obtained under controlled conditions at 20 °C and 25 °C for 24 h, respectively.

Figure 1 shows representative images of whole-wheat bread (CB) used as control and sourdough-substituted breads. The substitution of USWF with 20% of SD improved the specific bread volume (SBV) by 30–40% compared to the CB (Table 4). Conversely, the combination of both bioprocesses, sprouting and sourdough fermentation, did not present a synergistic effect. The increase in the SBV of the sourdough-substituted breads could be attributed to several factors. These include gluten expansion due to primary proteolysis by endogenous proteases synthesized during wheat sprouting and secondary proteolysis by LABs metabolism [6], acidification caused by LABs [32], and increased viscosity resulting from the rise in water-extractable arabinoxylans content in SD samples during fermentation [29]. Several authors [8,36] found a link between an increase in the content of water-extractable arabinoxylans during sourdough fermentation and an increase in the specific volume of wheat bread due to their ability to create a viscous environment and optimize the gluten network in the dough.

SB (SWF20) and SB (SWF25) exhibited higher crust browning index (Table 4). Considering that SWFs and their corresponding sourdoughs exhibited higher reducing sugars content and free amino acids compared to USWF, these compounds, being key precursors of Maillard reactions, may have contributed to crust browning during baking through the formation of colored melanoidins [37]. No significant differences were observed in the colorimetric parameters a* and b* of the crust or crumb (Table 4).

**Figure 2:**
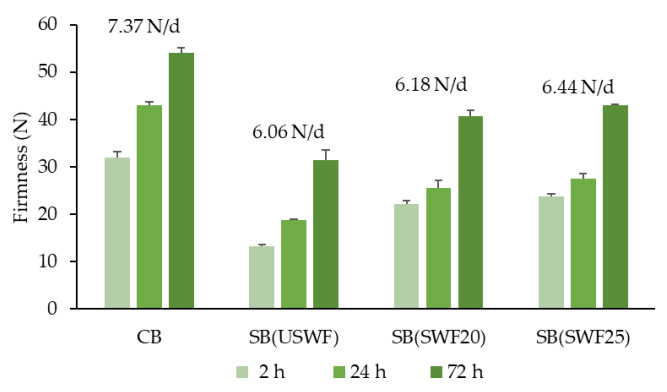
Crumb firmness of different breads 2, 24, and 72 h after baking. The firmness ratio is presented.

The initial firmness (2 h after baking) of the SB (USWF) decreased by 60% compared to CB (Figure 2). It was also observed that substituting 20% of the USWF with SD (SWFs) resulted in whole-wheat breads with a softer crumb than the CB, showing a 20% to 30% reduction in firmness. No synergistic effect was observed when combining sprouted whole-wheat flour and sourdough fermentation. After 72 h of storage at room temperature in sealed polyethylene bags, the crumbs of SB (USWF) and SB (SWFs) were significantly softer compared to the CB crumbs (Figure 2). The lower firmness observed at 24 h and 72 h of storage in the sourdough-substituted whole-wheat breads could be attributed to the larger volume of the bread pieces. Additionally, sourdough-substituted breads exhibited a reduced firming rate (Figure 2). Adhesiveness and springiness remained unchanged throughout the storage period, with no significant differences among samples (Table S1). After 3 days, sourdough-substituted breads exhibited lower gumminess and chewiness values compared to the CB (Table S1). In general, the extended shelf life of the sourdough-substituted breads could also be attributed to the significant role of amylolytic and proteolytic enzymes produced by LABs during the fermentation process [38].

### 3.5. Volatile Organic Compound Profile of Breads

Aroma, a key attribute for bread quality and consumer acceptance, is affected by both the fermentative microbiota and the raw materials used [39]. Substituting 20% of USWF with SD (USWF) or SD (SWF25) significantly impacted the aroma profiles. Table 5 shows the volatile organic compounds (VOCs) with relative peak areas above 0.01%. A total of 36 VOCs were identified. While SB (USWF) and SB (SWF25) shared similar VOC profiles, both differed from the CB, exhibiting a more diverse volatile spectrum. However, the impact of these compounds on final aroma depends largely on their individual odor activity values (OAVs) [40]. In CB, 26 compounds accounted for 91% of the total chromatogram area, compared to 32 (90%) in SB (USWF), and 36 (94%) in SB (SWF25) (Table 5).

Overall, the more diverse VOC profile found in sourdough-substituted breads may result from (i) extensive protein hydrolysis due to activation of wheat endoproteases at low pH, producing amino acids; (ii) the release of bound polyphenols; (iii) strong acidification; and (iv) the synthesis of aroma precursor compounds [40,41,42].

**Table 5:** Comparison of volatile organic compound profile identified by GC/MS analysis of control bread and sourdough-substituted breads.

Compounds	RT (min)	Odor Description *	Relative Peak Area (%)		
			CB	SB (USWF)	SB (SWF25)
Alcohols			83.11 ± 0.23 a	81.46 ± 4.75 a	87.61 ± 0.16 a
Ethanol	3.23	Strong, alcoholic	70.31 ± 2.56 a	71.13 ± 7.24 a	77.32 ± 0.59 a
2-methyl-1-propanol	6.54	Alcoholic, wine-like, malty	2.22 ± 0.07 a	1.13 ± 0.21 c	1.76 ± 0.00 b
2-methyl-butanol	9.87	Alcoholic, malty	1.76 ± 0.47 a	1.14 ± 0.25 a	1.07 ± 0.29 a
3-methyl-butanol	9.94	Balsamic, alcoholic, malty	5.48 ± 1.37 a	4.62 ± 1.04 a	4.77 ± 0.07 a
1-pentanol	10.9	Fruity	0.05 ± 0.01 a	0.03 ± 0.00 a	0.04 ± 0.02 a
1-hexanol	13.52	Grassy, woody, flowery	0.66 ± 0.20 a	0.43 ± 0.01 a	0.33 ± 0.14 a
1-heptanol	16.56	Alcoholic, green, citric	nd	nd	0.01 ± 0.00
2-furanmethanol	20.08	Alcoholic, honey, sweet	0.07 ± 0.04 a	0.09 ± 0.02 a	0.03 ± 0.01 a
z-4-decen-1-ol	22.42	Alcoholic, herbal	0.04 ± 0.02 a	0.06 ± 0.02 a	0.03 ± 0.01 a
Benzyl alcohol	23.56	Alcoholic, pleasant aroma	nd	0.02 ± 0.00 a	0.01 ± 0.00 b
2-phenyl-ethanol	23.94	Flowery, fermented yeast	2.52 ± 0.25 a	2.80 ± 0.97 a	2.25 ± 0.03 a
Aldehydes			1.38 ± 0.05 a	0.48 ± 0.17 b	0.61 ± 0.22 b
Acetaldehyde	1.74	Aldehydic, fruity	0.29 ± 0.06 a	0.05 ± 0.04 b	0.11 ± 0.02 b
2-methyl-propanal	2.09	Aldehydic, almond, malty	0.07 ± 0.00 a	0.04 ± 0.02 a	0.06 ± 0.03 a
2-methyl-butanal	2.82	Roasty, malty	0.09 ± 0.01 a	0.03 ± 0.01 a	0.06 ± 0.04 a
3-methyl-butanal	2.88	Roasty, malty	0.08 ± 0.01 a	0.08 ± 0.01 a	0.18 ± 0.13 a
Hexanal	5.90	Aldehydic, herbal, grassy	0.38 ± 0.03 a	0.13 ± 0.04 b	0.13 ± 0.03 b
Benzaldehyde	18.08	Aldehydic, caramel, almond	0.47 ± 0.03 a	0.14 ± 0.06 b	0.07 ± 0.01 b
Esters			0.87 ± 0.02 b	1.63 ± 0.15 a	1.38 ± 0.04 a
Ethyl acetate	2.59	Fruity, sweet, grassy, green	0.23 ± 0.04 b	0.08 ± 0.00 b	0.57 ± 0.10 a
3-methylbutyl acetate	6.86	Fruity	nd	0.23 ± 0.08 a	0.06 ± 0.04 b
Ethyl hexanoate	9.54	Fruity, apple peel-like	nd	0.08 ± 0.02 a	0.09 ± 0.03 a
Ethyl heptanoate	12.09	Fruity, grapes	nd	nd	0.04 ± 0.01
Ethyl octanoate	15.40	Fruity, sweet, fresh	0.35 ± 0.02 b	0.57 ± 0.12 a	0.27 ± 0.10 b
Ethyl nonanoate	18.05	Fruity	nd	0.18 ± 0.08 a	0.09 ± 0.05 a
2-phenylethyl acetate	22.73	Flowery	0.29 ± 0.04 b	0.50 ± 0.05 a	0.26 ± 0.03 b
Carboxylic acids			1.97 ± 0.06 b	4.26 ± 0.77 a	3.33 ± 1.26 a
Acetic acid	17.27	citric, flowery, pungent	1.56 ± 0.02 b	3.58 ± 0.97 a	3.02 ± 1.28 a
Hexanoic acid	23.29	Fatty, cheesy	0.41 ± 0.08 a	0.64 ± 0.19 a	0.29 ± 0.01 a
Heptanoic acid	24.46	Fatty, cheesy	nd	0.04 ± 0.01 a	0.02 ± 0.00 b
Heterocyclic compounds			0.40 ± 0.11 a	0.41 ± 0.07 a	0.21 ± 0.07 a
2-pentylfuran	9.24	Flowery, fruity	0.10 ± 0.07 a	0.10 ± 0.05 a	0.04 ± 0.02 a
Furfural	16.92	Almond, roasty, sweet	0.07 ± 0.02 a	0.12 ± 0.01 a	0.06 ± 0.05 a
Maltol	24.63	Sweet, caramel, fruity	nd	0.02 ± 0.00 b	0.03 ± 0.01 a
γ-nonanolactone	25.21	Fruity, sweet	0.23 ± 0.06 a	0.14 ± 0.05 a	0.07 ± 0.00 a
2-acetylpirrole	24.68	Nuts, cracker-like	nd	0.04 ± 0.02 a	0.03 ± 0.00 b
Ketones			0.46 ± 0.15 a	0.28 ± 0.02 a	0.22 ± 0.05 a
Acetoin	12.18	Butter, yogurt, cream	0.46 ± 0.15 a	0.28 ± 0.02 a	0.21 ± 0.06 a
Geranyl acetone	23.12	Flowery	nd	nd	0.01 ± 0.00

RT, retention time. Values followed by different lowercase letters within a row indicate significant differences between bread samples according to the DGC test (*p* ≤ 0.05). CB, control bread; SB (USWF) sourdough-substituted bread made with 20% of SD (USWF); SB (SWF25), sourdough-substituted bread made with 20% of SD (SWF25). nd, not detected. * Odor description as reported in the literature [40,42,43].

**Figure 3:**
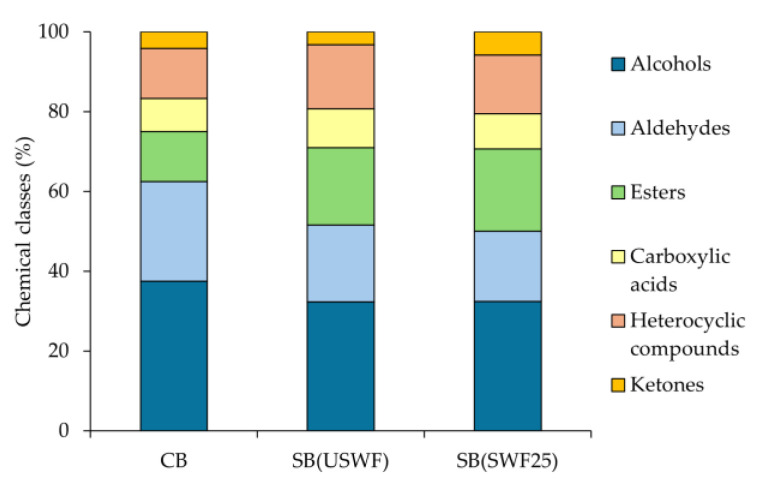
Relative abundance of volatile compound chemical classes of whole-wheat breads. CB, control bread; SB (USWF), sourdough-substituted bread made with 20% of SD (USWF); SB (SWF25), sourdough-substituted bread made with 20% of SD (SWF25).

The major chemical classes detected in whole-wheat breads were alcohols (predominant), followed by aldehydes and esters (Figure 3). Carboxylic acids, heterocyclic compounds, and ketones were present in smaller amounts. Ethanol, the main product of yeast fermentation, was the most abundant VOC, comprising ~70–75% of the total chromatographic area. *Saccharomyces cerevisiae*, which converts up to 95% of fermentable sugars into CO_2_ and ethanol, is mostly evaporated during baking [40].

During sourdough fermentation, yeast cells catalyze reactions between fatty acid (C6–C10) acetyl-CoA derivatives and alcohols (mainly ethanol), leading to the formation of a few esters [44]. In sourdough-substituted breads, esters such as 3-methylbutyl acetate, ethyl hexanoate, ethyl heptanoate, and ethyl nonanoate were detected. These esters are important for bread aroma, providing pleasant, sweet, and fruity notes [40]. An increase in the relative peak area of ethyl acetate and ethyl heptanoate, was observed in SB (SWF25).

Sourdough-substituted breads also contained heterocyclic compounds such as 2-acetylpyrrole and 3-hydroxy-2-methyl-4-pyrone (maltol), which are considered key crust aroma contributors due to their high OAVs [45,46].

Aldehydes such as acetaldehyde, hexanal, and benzaldehyde showed reduced peak areas in sourdough-substituted breads compared to CB. As hexanal is formed via lipoxygenase-mediated lipid oxidation, this suggests that increased yeast activity in sourdough may reduce oxygen availability for this reaction [40]. Although sprouting increases lipase activity [25], the formation of hexanal appears limited. Given its high OAV and association with undesirable aromas [44], reducing hexanal through sourdough addition may improve sensory acceptance.

Controlled sprouting has also been reported to modify bread flavor by enhancing sweetness and reducing bitterness [47]. In SB (SWF25), some VOCs, such as ethyl heptanoate (ester), heptanol (alcohol), and geranyl acetone (ketone), were detected, but they were not present in either the CB or the SB (USWF). Although these compounds have low OAVs [43,44], they are associated with pleasant aromas [48], which may represent a potential advantage of using sourdough made from sprouted whole-wheat flour.

Overall, the combined use of sourdough fermentation and sprouted whole-wheat flour not only diversifies the volatile profile of bread, but also reduces undesirable compounds, potentially enhancing both aroma complexity and consumer acceptance.

## 4. Conclusions

The use of sprouted whole-wheat flours as a fermentation substrate resulted in greater acidification and modification of macronutrients which could be beneficial for the nutritional aspects and technological quality of bread. These findings suggest that combining sprouting and fermentation may enhance protein digestibility, mineral bioavailability, and glycemic response compared to bread made exclusively with unsprouted, unfermented whole-wheat flour. Nevertheless, further research is required to confirm the potential nutritional and health benefits. The application of spontaneous sourdough fermentation proved to be an effective strategy for improving the technological quality of whole-wheat bread. Substituting 20% of the whole-wheat flour in the formulation with sourdough resulted in breads with greater specific volume and a softer crumb, both immediately after baking and after three days of storage, compared to breads made exclusively with whole-wheat flour. Additionally, sourdough-substituted bread made with sprouted whole-wheat flour exhibited a rich profile of volatile organic compounds associated with pleasant aromas, which may contribute to increased consumer acceptance.

## Figures and Tables

**Table 1:** Chemical composition of flour and sourdough samples.

Samples	α-Amylase Activity (AU/g db)	Reducing Sugar (g/100 g db)	Water-Extractable Arabinoxylans (g/100 g db)	Free Amino Acid Groups (µmol Serine/mg of Protein db)
USWF	0.45 ± 0.03 c	1.28 ± 0.50 b	0.86 ± 0.02 b	0.21 ± 0.00 c	
SWF20	11.81 ± 0.01 a	5.37 ± 0.89 a	1.48 ± 0.20 a	0.39 ± 0.01 a	
SWF25	12.19 ± 0.55 a	5.27 ± 0.43 a	1.79 ± 0.08 a	0.36 ± 0.01 b	
SD (USWF)	0.14 ± 0.01 A	5.75 ± 0.52 B	1.38 ± 0.07 B	0.82 ± 0.01 C	
SD (SWF20)	0.16 ± 0.02 A	6.44 ± 0.61 B	1.95 ± 0.09 A	0.92 ± 0.03 B	
SD (SWF25)	0.12 ± 0.01 A	8.72 ± 0.52 A	2.04 ± 0.09 A	1.11 ± 0.01 A	

Values followed by different lowercase letters within a column indicate significant differences between flours, while values followed by different uppercase letters indicate significant differences between sourdoughs, according to the DGC test (*p* ≤ 0.05). USWF, unsprouted whole-wheat flour; SWF20 and 25, sprouted whole-wheat flour obtained after 24 h of sprouting at 20, and 25 °C, respectively; SD (USWF)**,** sourdough made with USWF; SD (SWF20 and 25), sourdough made with SWF20 and 25, respectively.

**Table 2:** Determination of pH, total titratable acidity, and microbiological analysis in ripe sourdoughs at the start of fermentation and after 7 days of daily refreshment.

Time	Sample	pH	TTA (mL NaOH/g)	ΔpH	Yeast (log CFU/mL)	LAB (log CFU/mL)
t0	SD (USWF)	6.02 ± 0.12 a	3.75 ± 0.07 b	-	2.36 ± 0.08 b	3.15 ± 0.07 b
	SD (SWF20)	5.50 ± 0.04 b	12.15 ± 0.07 a	-	4.13 ± 0.17 a	6.21 ± 0.19 a
	SD (SWF25)	5.45 ± 0.02 b	10.00 ± 0.14 a	-	3.96 ± 0.11 a	6.24 ± 0.24 a
t7	SD (USWF)	3.74 ± 0.01 B	17.00 ± 0.00 A	2.27 ± 0.11 A	6.69 ± 0.06 B	8.25 ± 0.07 B
	SD (SWF20)	3.80 ± 0.01 A	17.50 ± 0.71 A	1.71 ± 0.02 B	6.90 ± 0.03 A	8.39 ± 0.04 A
	SD (SWF25)	3.78 ± 0.01 A	18.00 ± 0.00 A	1.67 ± 0.03 B	6.27 ± 0.08 C	8.51 ± 0.05 A

Values followed by different lowercase letters within a column indicate significant differences between sourdoughs at t0, while values followed by different uppercase letters indicate significant differences between sourdoughs at t7, according to the DGC test (*p* ≤ 0.05). SD (USWF), sourdough made with unsprouted whole-wheat flour; SD (SWF20 and 25), sourdough made with sprouted whole-wheat flour obtained after 24 h of sprouting at 20 and 25 °C, respectively.

**Table 3:** Free phenolic content, antioxidant capacity and phytic acid content of flour and sourdough samples.

Sample	TPC (mg GAE/100 g db)	FRAP (µmol TE/g db)	ABTS˙+ (µmol TE/g db)	PA (mg PAP/g db)
USWF	50.28 ± 1.70 c	20.63 ± 0.73 b	6.16 ± 0.73 b	19.93 ± 0.07 a
SWF20	86.47 ± 0.96 b	27.74 ± 0.10 a	9.66 ± 1.67 a	18.25 ± 0.17 b
SWF25	137.78 ± 0.46 a	26.51 ± 0.53 a	11.12 ± 0.18 a	18.29 ± 0.26 b
SD (USWF)	129.67 ± 0.16 B	25.85 ± 1.45 B	17.10 ± 0.64 A	0.92 ± 0.06 A
SD (SWF20)	160.15 ± 17.83 A	31.50 ± 0.93 A	21.11 ± 1.49 A	0.96 ± 0.09 A
SD (SWF25)	149.40 ± 5.42 A	30.43 ± 0.53 A	19.77 ± 0.47 A	1.00 ± 0.09 A

Values followed by different lowercase letters within a column indicate significant differences between flours, while values followed by different uppercase letters indicate significant differences between sourdoughs, according to the DGC test (*p* ≤ 0.05). USWF, unsprouted whole-wheat flour; SWF20 and 25, sprouted whole-wheat flour obtained after 24 h of sprouting at 20 and 25 °C, respectively; SD (USWF), sourdough made with USWF; SD (SWF20 and 25), sourdough made with SWF20 and 25, respectively; GAE, gallic acid equivalent; TE, Trolox equivalent.

**Table 4:** Technological parameters of whole-wheat breads.

Parameters	CB	SB (USWF)	SB (SWF20)	SB (SWF25)
Specific bread volume (mL/g)	1.69 ± 0.04 b	2.34 ± 0.09 a	2.22 ± 0.02 a	2.23 ± 0.01 a
Crust				
Browning (100-L*)	38.56 ± 0.34 b	41.09 ± 0.81 b	43.25 ± 1.48 a	44.57 ± 1.67 a
a*	10.84 ± 1.05 a	12.77 ± 0.89 a	11.72 ± 0.83 a	11.29 ± 1.43 a
b*	24.91 ± 0.72 a	22.64 ± 2.17 a	25.53 ± 2.18 a	24.82 ± 1.53 a
Crumb				
Browning (100-L*)	43.57 ± 0.05 a	45.01 ± 1.73 a	42.96 ± 2.31 a	43.42 ± 1.92 a
a*	7.40 ± 0.10 a	7.05 ± 0.22 a	6.79 ± 0.42 a	8.00 ± 0.37 a
b*	21.94 ± 0.21 a	22.17 ± 0.78 a	21.86 ± 0.49 a	22.72 ± 0.16 a

Different letters in the same row indicate significant differences according to the DGC test (*p* ≤ 0.05). CB, control bread; SB (USWF), sourdough-substituted bread made with 20% sourdough from unsprouted whole-wheat flour; SB (SWF20 and SWF25), sourdough-substituted bread made with 20% sourdough from sprouted whole-wheat flours obtained under controlled conditions at 20 °C and 25 °C for 24 h, respectively.

## Data Availability

The raw data supporting the conclusions of this article will be made available by the authors on request.

## Supplementary Materials

The following supporting information can be downloaded at https://www.mdpi.com/article/10.3390/foods14162805/s1, Table S1: Texture profile of bread samples

## Abbreviations

The following abbreviations are used in this manuscript:

USWF	Unsprouted whole-wheat flour
SWF	Sprouted whole-wheat flour
SWF20	Sprouted whole-wheat flour obtained after sprouting at 20 °C
SWF25	Sprouted whole-wheat flour obtained after sprouting at 25 °C
SD (USWF)	Sourdough made with USWF
SD (SWF20)	Sourdough made with SWF20
SD (SWF25)	Sourdough made with SWF25
CB	Control bread made with USWF
SB (USWF)	Sourdough bread made with USWF
SB (SWF20)	Sourdough bread made with SWF20
SB (SWF25)	Sourdough bread made with SWF25
DoS	Degree of sprouting
db	Dry basis
LAB	Lactic acid bacteria
TTA	Total titratable acidity
DNS	3,5-dinitrosalicylic acid
AU	α-amylase unit
OPA	*o*-phthalaldehyde
PAP	Available phosphorus phytate
GAE	Gallic acid equivalent
TE	Trolox equivalent
SBV	Specific bread volume
VOC	Volatile organic compound
TPA	Texture profile analysis
OAV	Odor activity value
SPME	Solid phase extraction
ANOVA	Analysis of variance
