# Supplementary material for: Chemical and Nutritional Characterization of Sourdoughs Made with Sprouted and Unsprouted Whole-Wheat Flour and Their Effects on the Technological Quality of Bread
Source: Foods. 2025 Aug 13;14(16):2805. doi: 10.3390/foods14162805 (PMC12385715; doi:10.3390/foods14162805)
Supplement: Supplementary file 1 [file foods-14-02805-s001.zip › foods-3784783 - Supplementary File.pdf]

Supplementary Materials

Table S1. Texture profile of bread samples

Parameters	Storage time (h)	CB	SB(USWF)	SB(SWF20)	SB(SWF25)
Adhesiveness	2	0.00018 ± 0.00 a	0.00018 ± 0.00 a	0.00020 ± 0.00 a	0.00020 ± 0.00 a
(J)	72	0.00017± 0.00 a	0.00020 ± 0.00 a	0.00017 ± 0.00 a	0.00017 ± 0.00 a
Springiness	2	0.97 ± 0.01 a	0.99 ± 0.00 a	0.96 ± 0.07 a	0.99 ± 0.01 a
	72	0.97 ± 0.01 a	0.96 ± 0.02 a	0.94 ± 0.05 a	0.91 ± 0.02 a
Gumminess	2	16.33 ± 0.83 a	7.22 ± 1.14 b	13.53 ± 1.43 a	11.07 ± 1.68 a
(N)	72	26.42 ± 2.67 a	16.63 ± 0.63 b	20.86 ± 2.24 b	18.09 ± 1.08 b
Chewiness	2	15.91 ± 0.73 a	7.18 ± 1.11 c	12.90 ± 0.45 b	10.98 ± 1.59 b
(N)	72	25.63 ± 2.17 a	16.04 ± 0.88 b	19.59 ± 0.97 b	16.52 ± 1.41 b

Different letters in the same row indicate significant differences according to the DGC test ($p \leq 0.05$). CB, control bread; SB(USWF), sourdough-substituted bread made with 20% sourdough from unsprouted whole-wheat flour; SB(SWF20 and SWF25), sourdough-substituted bread made with 20% sourdough from sprouted whole-wheat flours obtained under controlled conditions at 20 °C and 25 °C for 24 h, respectively. J, joule; N, newton.
